# Physicochemical stability of bortezomib solutions for subcutaneous administration

**DOI:** 10.1038/s41598-024-58473-1

**Published:** 2024-04-18

**Authors:** Ángela Gómez, Mª Cristina Benéitez García, Nélida Barrueco, Mª Amparo Lucena-Campillo, Elena López-Lunar, Benito García-Díaz, Marta Vicario-de-la-Torre, Ismael Escobar-Rodríguez, María Esther Gil-Alegre

**Affiliations:** 1 Department of Pharmaceutical and Food Technology, Complutense University of Madrid, 28040 Madrid, Spain; 2 Department of Basic Health Sciences, Area of Pharmacy and Pharmaceutical Technology, Faculty of Health Sciences, Rey Juan Carlos University, Madrid, Spain; 3 Pharmacy Service, Infanta Leonor Hospital, Madrid, Spain; 4 Severo Ochoa Hospital, Madrid, Spain; 5 Department of Pharmacy Jose Germain Psychiatric Institute. Department of Pharmacy and Pharmaceutical Technology, Complutense University of Madrid, Madrid, Spain; 6 Pharmacy Service, Severo Ochoa Hospital, Madrid, Spain

**Keywords:** Bortezomib, Physicochemical stability, Ready-to-administer subcutaneous injection, Stability study protocol, Prefilled syringes, And open vials, Health care, Oncology, Materials science

## Abstract

For the majority of cytotoxic drug preparations, such as bortezomib, the unit dose information is not available. In addition, there is a lack of information on the physicochemical stability of the pharmaceutical preparation after opening; this information is crucial for its administration to patients in successive visits, and the per-patient cost can be affected. The purpose of our proposed physicochemical stability study is to determine the shelf life of the reconstituted liquid product under refrigeration and clinical practice conditions. This evaluation was extended to both vials and ready-to-use syringes prefilled with the contents of the open vial. The stability test design includes the specified storage conditions and the critical physicochemical parameters of reconstituted injectable bortezomib. Furthermore, this approach includes the determination of impurities, the monitoring of the purity of the mean peak using a photodiode array, the control of the mass balance, the monitoring of subvisible particles using a laser diffraction analyser, and the setting of stability specifications. For the chemical stability study, the amount of bortezomib and its degradation products were determined using a stability-indicating HPLC method. The physical inspection of the samples was performed throughout the stability study, and their pH values were also monitored. Bortezomib (2.5 mg/mL) in 0.9% sodium chloride remained stable for 7 days when stored in both polypropylene syringes and vials at 5 ± 3 °C (refrigeration) and shielded from light. Additionally, it exhibits stability for 24 h under storage conditions simulating clinical use (20–30 °C and protected from light). The proposed protocol provides the stability in the vials once reconstituted and in prefilled refrigerated syringes; this protocol can be used to reduce waste and increase cost savings.

## Introduction

Surgery, radiation therapy, chemotherapy, hormone therapy, and immune therapy are employed as cancer treatment methods. Unlike surgery and radiation therapy, chemotherapy has a systemic impact. It is considered one of the most effective treatment strategies available to physicians, allowing personalized doses of the cytostatic drugs according to cancer type and stage as well as patient age and health^1^.

In the majority of cytotoxic drug preparations, such as bortezomib, appropriate unit doses information is not available. In addition, information pertaining to the physicochemical stability of the pharmaceutical preparation (vials) after the vials are opened is crucial for its administration to patients in successive visits, and the per-patient cost can be affected.

Stability studies of cytotoxic drug preparations^2–5^ are clearly needed since the stability information provided by manufacturers can be inconsistent for the same active substance under the same conditions (dilution and excipients). This can undoubtedly create confusion in clinical practice, especially when considering the suitability of diluted vial contents or prefilled syringes for administration. These studies are for bortezomib.

It has been frequently observed that the shelf life of these pharmaceutical forms is hours^4–21^.

Even Jean-Daniel Hecq^22^ noted that “Several reference works are available to help the hospital pharmacist with this research. However, reading these different sources can make you discover conflicting data,” due to different analysis conditions.

The purpose of our proposed physicochemical stability study is to determine the shelf life of the reconstituted liquid product under refrigeration and clinical practice conditions. This evaluation was extended to both vials and ready-to-use syringes prefilled with the contents of the open vial. This knowledge could allow personalized doses of cytostatic drugs to be prepared in advance before administration. This potentially helps to improve workflow in hospital pharmacies, saving time in outpatient departments and reducing the costs of medicines intended for a single patient.

This stability test design sets the specified storage conditions and the critical physicochemical parameters for the stability of reconstituted injectable cytotoxic drug products.

Furthermore, this approach includes the determination of the degradation products, the monitoring of the purity of the mean peak using a photodiode array, the control of the mass balance, the monitoring of subvisible particles using a laser diffraction analyser, the setting of stability specifications and the statistical data treatment. The protocol meets the technical requirements of pharmaceuticals for human use^23–26^.

Additionally, our proposed protocol could help to increase the shelf life and clinical condition stability of reconstituted bortezomib in vials and syringes at the concentrations needed for subcutaneous administration. This is particularly relevant because its approval is more recent than its intravenous approval, and limited stability information is currently available.

Bortezomib is an inhibitor of the 26S proteasome in mammalian cells It received US Food and Drug Administration (FDA) approval in May 2003 and European Medicines Agency (EMA) approval in April 2004 for the treatment of multiple myeloma (FDA, EMA) and mantle cell lymphoma (FDA).

For its application, after the bortezomib in Velcade^®^ was reconstituted with 3.5 mL of 0.9% sodium chloride solution (with final bortezomib concentration of 1 mg/mL), it needed to be intravenously administered within 8 h at 25 °C when stored in the original vial and/or syringe^27^.

In January 2012 and October 2012, respectively, the FDA^27^ and EMA^28^ authorized a new route of administration (subcutaneous) for bortezomib in multiple myeloma treatment. Subcutaneous bortezomib provides noninferior efficacy with a lower incidence of relevant adverse effects, such as peripheral neuropathy^29^, than standard intravenous administration. For subcutaneous administration, each vial of bortezomib was reconstituted with 1.4 mL of 0.9% sodium chloride to produce a final concentration of 2.5 mg/mL.

Even though the vials sold for both routes of administration are the same, the difference in the final concentration after reconstitution in the subcutaneous version means that the stability data for the 1 mg/mL solution cannot be extrapolated. Hence, hospital pharmacists are required to follow the stability guidelines provided by the manufacturer, always applying their training and experience or checking published data^22^.

Considering the information provided by the manufacturers, the following two main situations for bortezomib have been found (information from the FDA^27^ and/or EMA^28^ and/or AEMPS^30^): different shelf life for each of the two different reconstituted concentrations based on the route of administration or different shelf life and different storage conditions once bortezomib is reconstituted. In addition, the final concentration of bortezomib is not always specified.

In this context, our proposed protocol can be used to determine the physical and chemical stability of bortezomib reconstituted with 1.4 mL of sterile 0.9% sodium chloride solution (concentration 2.5 mg/mL) in original manufacturer vials and prefilled syringes.

## Results

### Chemical stability study

**Figure 1:**
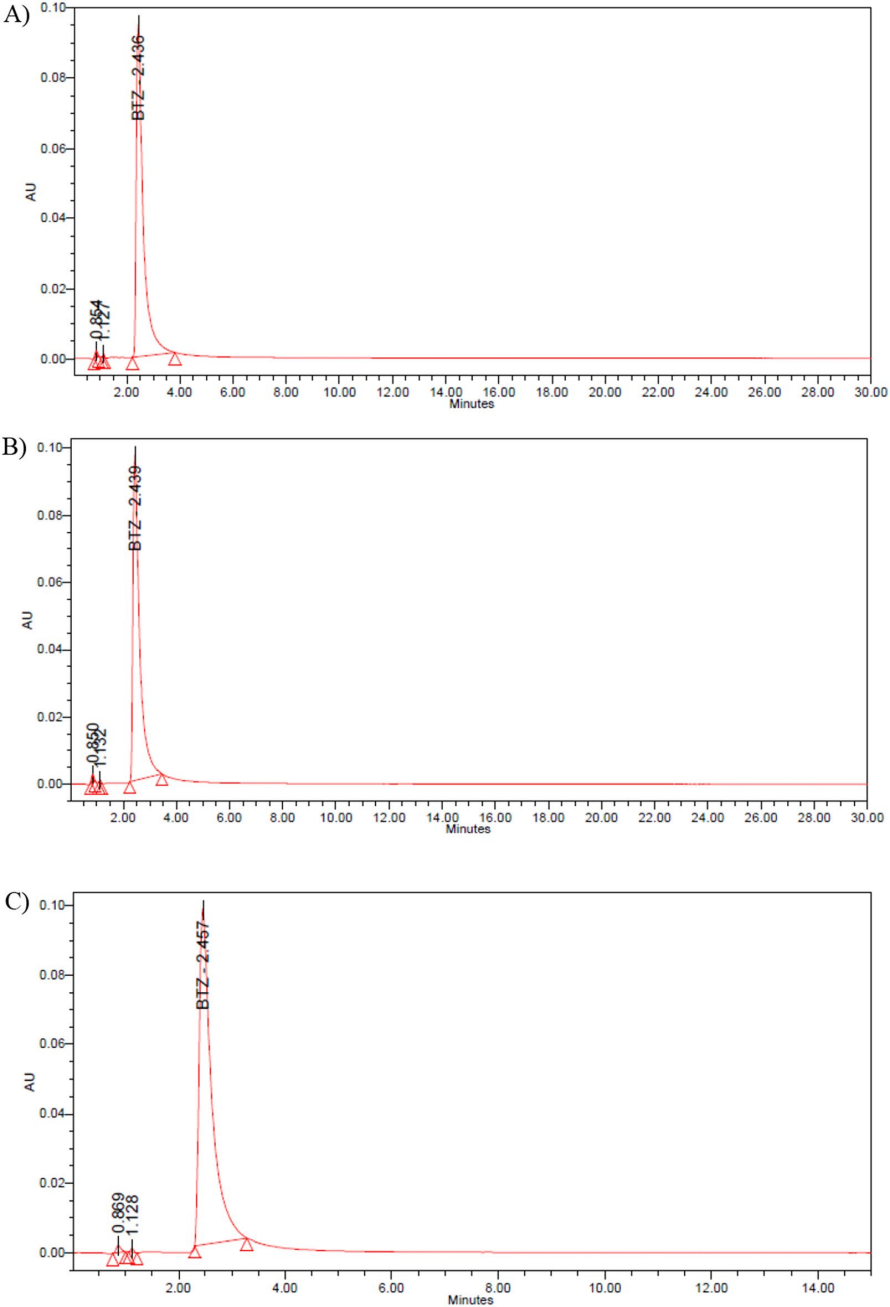

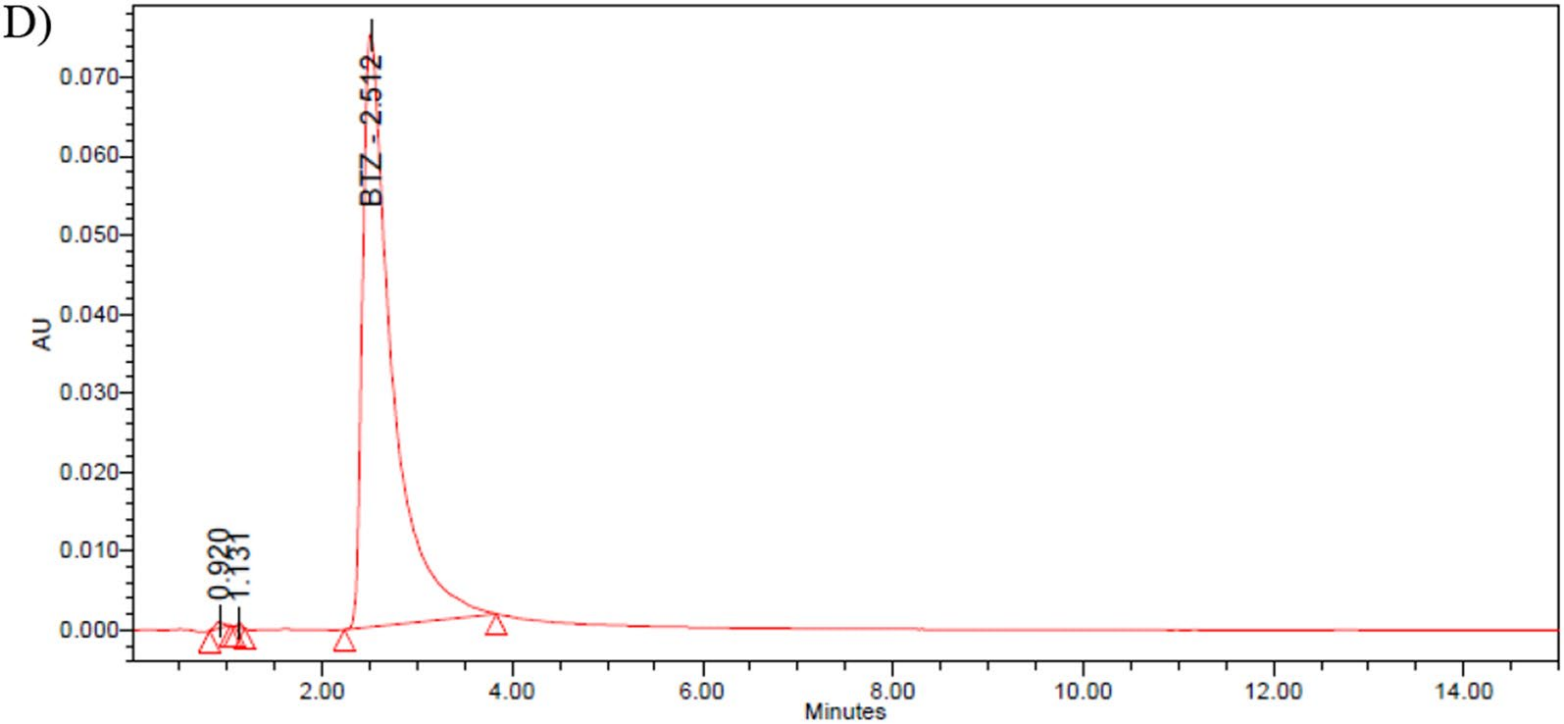
Chromatograms of the bortezomib reference solution (125 µg/mL) (**A**) and bortezomib test solution (125 µg/mL) at the initial time (**B**) and after 10 days at 5 ± 3 °C and in the dark (syringe (**C**), vial (**D**)).

The obtained chromatograms of bortezomib are shown in Fig. 1. No overlap was observed with the main peak for bortezomib. The main peak corresponding to bortezomib (eluting at 2.4 min) remained unaffected by the degradation products (eluting before the main peak: 0.8, 1.1 and 1.6 min).

System suitability analysis, in accordance with US Pharmacopeia (USP) guidelines, showed that the resolution between drug substance and diastereomer peaks was ≥ 2.0, the relative standard deviation RSD of peak area responses in 6 consecutive injections of the working standard (125 µg/mL) was ≤ 2.0%, and the percentage difference in average response factors between working and check standard injections (50, 125 and 175 µg/mL) was ≤ 1.0%.

Purity evaluation of the main peak of bortezomib using a diode array detector (PAD), also known as diode array detector (DAD), revealed values exceeding 99% in all the measurements.

The linearity of the method was assessed. The 6-point curve was linear over the range of concentrations from 50 µg/mL to 175 µg/mL. A significant correlation for the calibration curve was found: correlation coefficient > 0.987, ANOVA _regression_ α < 0.05_,_ ANOVA_linear fit_ α > 0.05). The intraday and interday RSDs were lower than 1.5% (n = 6, same day) and 2.2% (n = 2 × 3 days = 6), respectively. After determining the RSD of the assay, a power calculation indicated that duplicate analysis of samples could be used to distinguish between concentrations that differed by at least 0.5%^31^.

In the chemical stability study, a mass balance assessment was conducted, revealing values closely aligning with 100% of the initial bortezomib value. Considering the margin of analytical error, the values of mass balance were determined to be acceptable. Thus, the experimental values were suitable for determining the chemical stability of bortezomib.

ANOVA revealed significant differences in the percentage remaining in vials or syringes stored under refrigerated and dark conditions throughout the 10-day study period (Table 1). Under these conditions (5 ± 3 °C and darkness), the lower limit of the 95% confidence interval of concentration remaining was greater than 95% of the initial (Day 0) concentration for 7 days.

**Table 1:** Stability of bortezomib (2.5 mg/mL) in storage containers (vials and syringes) at 5 ± 3 °C in the dark (all data are presented as the mean ± S.D. of test results for all analysed samples from 3 different vials or syringes and 2 samples withdrawn from each preparation (n = 6)).

Sample type	Initial drug concentration (mg/mL) Mean ± S.D	Drug concentration remaining (%) Mean ± S.D				
		1 day	3 days	4 days	7 days	10 days
Glass vial	2.46 ± 0.01	99.75 ± 0.26	97.59 ± 0.54	98.05 ± 0.35	95.89 ± 0.33	91.56 ± 0.60
Polypropylene syringes	2.45 ± 0.04	100.76 ± 0.24	98.02 ± 0.62	97.62 ± 0.68	95.84 ± 0.41	91.02 ± 0.53

The nominal concentrations of bortezomib did not differ between the syringe and vial containers, and both met the criteria for stability when stored at 5 ± 3 °C and in darkness for 7 days (Table 1).

The initial concentration of bortezomib remained above 95% for 24 h in all evaluated vials and syringes under in-use clinical conditions and protected from light: 98.60% ± 1.29% in vials and 97.74% ± 1.63% in syringes (n = 6, 3 different vials or syringes and 2 samples were withdrawn from each preparation). The proposed in-use clinical period of the reconstituted product was confirmed in samples at the final point of the stability study: after 7 days of storage in a refrigerator and protected from light.

The degradation product levels remained below the reporting threshold^32^ at all points (3, 4 and 7 days) for Impurity 1 (retention time of 1.0 min) and Impurity 2 (retention time of 1.6 min) in both glass vials and polypropylene syringes at 5 ± 3 °C in the dark; values between 0.03 ± 0.001 and 0.04 ± 0.001% of the drug substance (mean ± standard deviation, n = 6) were obtained. Hence, the degradation products did not impose any limitations on the shelf life of the reconstituted subcutaneous bortezomib solution.

All samples met the acceptance criteria for appearance, physical attributes, and pH values. Under visual inspection, no colour changes or particles were detected in the solution. No particles ≥ 10 µm were detected using a laser diffraction particle size analyser, taking into account the limitation related to the unknown limit of detection.

**Figure 2:**
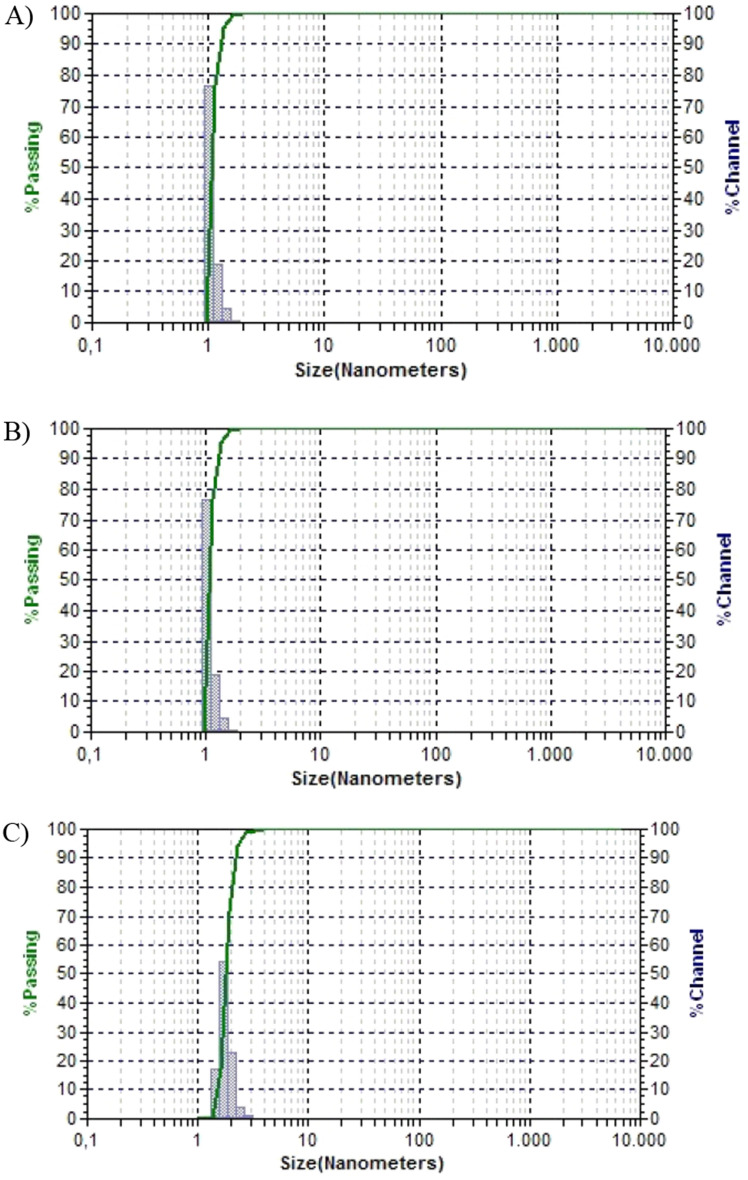
Particle size histograms of the subcutaneous bortezomib solution (2.5 mg/mL, vial) at time zero (**A**) at 24 h under in-use clinical conditions and protected from light (**B**) and at 10 days under refrigerated conditions (5 ± 3 °C and in darkness) (**C**).

Furthermore, when monitoring potential particle growth (Fig. 2), dynamic light scattering analysis revealed a shift in particulate size when the reconstituted preparations were stored in a refrigerator for 10 days (Fig. 2C). The results showed that the shifts in the particle sizes were acceptable, despite the lack of specifications for this type of modification since no particles were > 10 µm. The solution pH (initial pH = 5.77 ± 0.05) did not significantly change over the 10-day study period in the tested samples; a variation of less than one pH was obtained. The mean pH values of the samples from syringes or vials in darkness, refrigerated, and in-use clinical conditions, throughout the study, were 5.42 ± 0.11, 5.18 ± 0.09, and 5.44 ± 0.21, respectively.

## Discussion

The physicochemical stability protocol for the reconstituted bortezomib at a concentration of 2.5 mg/mL was successfully performed, utilizing both the original manufacturer vials and prefilled syringes.

Although the bortezomib stabilities in the original manufacturer vials and prefilled concentration (1 mg/mL in 0.9% sodium chloride) were already assessed in previous studies^27,28,33^, limited data was available for 2.5 mg/mL bortezomib in the same solution and containers. For this concentration, only one manufacturer^34^ and specific publications^3,35,36^ provided information, and a shelf life exceeding the manufacturer's recommendation was suggested.

The manufacturer stated that chemical and physical stability was demonstrated for 24 h post-opening, under conditions of 25 °C and normal indoor lighting, in the original vial and/or in a polypropylene syringe. This result aligned with our findings concerning clinical conditions of use. However, unlike our study, their study lacked stability data for prefilled syringes under different conditions.

Vigneron, J. et al.^3^ reported that bortezomib reconstituted at 2.5 mg/mL in 0.9% NaCl was stable for 30 days in glass vials at 2–8 °C; they referenced Espinosa, M. et al.^35^, who noted that "During the study period, the concentration in all study samples retained at least 88.18% of the initial concentration of bortezomib". This result was attained after 24 h in manufacturer vials stored at 4 °C in the dark. The report by Vigneron, J. et al.^3^ appeared to be inconsistent with the original work^35^.

Walker, S.E. et al.^37^ reported that bortezomib concentrations were considered "acceptable" or "within acceptable limits" if the lower limit of the 95% confidence interval of the remaining concentration exceeded 90% of the initial (Day 0) concentration (T-90_95% CI_). The justification for this 90% threshold (initial concentration) was not provided and was less rigorous than our threshold, and therefore, the 95% of the initial concentration was supported and justified in our study, since we used T-95_95% CI_ as a criterion for the acceptance of the results. Moreover, studies of the stability of these materials in prefilled syringes were exclusively performed only when the syringes were stored at 4 °C, and the data for prefilled syringes under clinical conditions or conditions of use are lacking.

In addition, in these stability studies, physical stability evaluation has relied primarily on visual inspection^38–40^, whereas in our study, monitoring the particle size of freshly reconstituted and stored reconstituted preparations (vials and syringes) is proposed. Moreover, laser diffraction was used for the determination of the potential particle growth, either independently or by employing dynamic light scattering (DLS) when particles ≥ 10 µm were not detected in the analysed samples. A slight increase in particle size was detected in samples stored in a refrigerator for 10 days. This was potentially caused by a precipitation phenomenon.

For storage conditions, the actual temperature should be monitored throughout a stability study^3,4,22^. However, few of these previous stability studies have shown fluctuations in the storage temperature. The determination of the thresholds tolerated under specific storage conditions is essential for defining accurate label storage statements and ensuring the shelf life of medicines. Consequently, the 21-day shelf life of bortezomib (3.5 mg in the manufacturer's vial) reconstituted with 1.4 mL of NS at 23 °C^36,37^ could only be applied to subcutaneous solutions of bortezomib kept at that exact temperature. Terms such as “ambient conditions” or “room temperature” need to be avoided^24^ to provide a direct link between the label storage statement and the demonstrated drug stability.

None of the stability studies adhered to harmonization achievements in the ICH Quality area for medicines of human use, such as the relevant thresholds for impurity testing, according to pivotal milestones^32^. Moreover, none of these stability studies monitored the mass balance^24^.

Investigations into the degradation of bortezomib under various stress conditions^35–37,41–46^ showed 5 known degradation products. Monitoring these degradation products is crucial because they are considered impurities in drug products^32^. Furthermore, the shelf life may be limited by the presence of a certain amount of these products. However, existing studies on bortezomib stability did not monitor these degradation products in reference to the regulatory limits of impurities. The stability period established in our study aligned with these limits for impurity levels in drugs.

In this study, the purity of the peak of bortezomib was evaluated using a photodiode array (PDA), and in all cases, the purity was greater than 99%. PDA is often used to determine the peak purity of a target compound. The absorbance spectra were compared at multiple points across the peak for similarities. A peak purity index was generated, which could indicate whether multiple compounds coeluted. The tailing factor was also monitored. This factor did not significantly change from the fresh to the degraded samples. For this reason, the tailing factor was found to be unrelated to the hidden breakdown products. Then, the other products were considered not hidden.

In conclusion, 2.5 mg/mL bortezomib in 0.9% sodium chloride remained stable for 7 days when stored in both polypropylene syringes and original manufacturer vials at 5 ± 3 °C (refrigeration) and protected from light. Additionally, 2.5 mg/mL bortezomib in 0.9% sodium chloride was stable for 24 h when stored in both polypropylene syringes and original manufacturer vials under storage conditions simulating clinical use (20–30 °C and protected from light).

A thorough physicochemical stability study protocol needs to determine the shelf life and in-use physicochemical stability (stability under clinical practice conditions) of 2.5 mg/mL bortezomib, which requires reconstitution prior to parenteral administration. Our proposed stability protocol provides stability in the original manufacturer vials once reconstituted, resulting in waste reduction and enhancing efficiency through cost savings. It also provides stability in the prefilled refrigerated syringes, facilitating the preparation of personalized doses in advance prior to administration, thereby improving workflow in hospital pharmacies and saving time in outpatient departments.

## Methods

### Chemical and physical stability design storage conditions

The storage conditions need to be specified and must meet the requirements^22–26^. According to climatic zones (WHO) and conditions recommended by the ICH guidelines, the proposed storage conditions for reconstituted cytotoxic drug liquid preparations include storage under “clinical conditions” for the chemical and physical “in-use stability” determination and storage in a refrigerator for the chemical and physical “shelf-life” determination.

In particular, the proposed storage conditions for the reconstituted cytotoxic drug liquid preparations under “clinical conditions,” “conditions of use” or “room temperature” are as follows: temperatures between 23 °C and 32 °C to for all the Climatic Zones (25 ± 2 °C for Climatic Zones I and II and 30 ± 2 °C for Zones III and IV; this temperature should be specified), humidities ranging from 30 to 45% relative humidity (RH) for the products packaged in semipermeable containers (e.g., prefilled syringes), and protection from light. The light conditions (diffuse daylight, room lighting) in an open laboratory environment are very different among all hospitals, even for the same hospital, and can cause serious light stability problems. To minimize these problems, the samples were protected from light.

The proposed storage conditions for reconstituted cytotoxic drug liquid preparations in a refrigerator are 5 °C ± 3 °C. The samples should not be frozen if the affects from the freezing–thawing process were not determined^4^.

**Figure 3:**
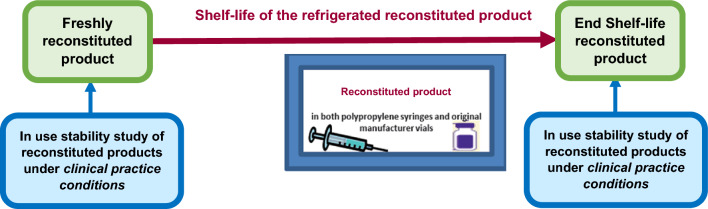
Chemical and physical in-use stability study for reconstituted cytotoxic drug preparations in both open vials and prefilled syringes from open vials. The chemical and physical stability of the reconstituted product under clinical practice conditions is measured not only for freshly prepared products but also at the end of the shelf-life of the refrigerated samples.

The chemical and physical in-use stability of the reconstituted product under clinical practice conditions should be checked not only for freshly prepared products but also at the end of the shelf-life of the refrigerated samples (Fig. 3).

More specifically, the stabilities of the products reconstituted with bortezomib for shelf-life determination were determined as follows: the syringes and vials were kept refrigerated, protected from light (light-proof plastic bags, Ultramedic, Miramed S.L., Barcelona, Spain, batch number 12008.00), and assayed at 0, 1, 3, 4, 7 and 10 days after preparation. For chemical and physical in-use stability determination simulating clinical conditions for the bortezomib-reconstituted product (clinical practice conditions), the syringes and vials were kept under clinical conditions (29 ± 2 °C, as worse case) and protected from light (light-proof plastic bags, Ultramedic, Miramed S.L., Barcelona, Spain, batch number 12008.00) and assayed at 12 and 24 h. Syringes were considered semipermeable containers, and the storage conditions of prefilled syringes also met the RH requirements (30 to 45% relative humidity).

### Chemical stability study

#### Drug content assay

The concentration of bortezomib in solution was determined using an HPLC system.^22^.

The HPLC assay was based on previously reported methods^35,41^.

All information to permit the replication of the analysis^22^ is provided below. The analysis was performed at room temperature (25 °C) using an HPLC instrument (Waters Milford, MA, USA) equipped with an autosampler (injection volume of 20 µL). The signal from the detector (2998 Photodiode Assay Detector, Waters, USA) working at 200–350 nm was recorded and integrated with a chromatography data system (Empower 3.6.1 Waters Corporation, Kyoto, Japan). A C18 (5 µm; 25 cm × 4 mm) column (LiChrospher^®^ 100 Supelco^®^ Analytical Products, Spain, Lot number: NF-21902) was used. The isocratic mobile phase consisted of a mixture of HPLC grade acetonitrile (HPLC grade, Panreac, Barcelona, Spain, Lot number: 0000341710) and water (40:60, v/v) at a flow rate of 1.5 mL/min.

A stock solution containing 2.5 mg/mL bortezomib was prepared using Velcade® (Janssen-Cilag Internacional, Beerse, Belgium, batch numbers GLZS500 and GLZS600) diluted with 0.9% sodium chloride (0.9% sodium chloride, USP, B. Braun Medical S.A., Rubí, Spain, batch number: 20FIA013) to yield concentrations of 50, 75, 100, 125, 150 and 175 µg/mL. The use of a drug product instead of a standard analyte was a limitation of the study.

Quantitation was performed by integrating the area under the curve with a chromatography data system (Empower 3.6.1.). The purity of the main peak was evaluated to confirm the absence of hidden compounds (2998 Photodiode Assay Detector, from 200 to 350 nm).

The system suitability analysis^47^ was performed according to the United States Pharmacopeia (USP) guidelines.

Reference standards were prepared for each test sample and injected for HPLC analysis (Waters, Milford, MA, USA, equipped with a photodiode array detector -PDA-). External standard injections (aqueous solution of caffeine at 0.02 mg/mL, LiChroTest^®^). Merck KGaA, Darmstadt, Germany, Batch number: 1191630001) were systematically included in the analysis to check the HPLC equipment.

A 50-µL volume was aseptically withdrawn from each syringe (Polypropylene syringes, 3 mL, BD Medical, Becton Dickinson, Franklin Lakes (NJ), USA, batch number 2304241 The syringe was provided with a subcutaneous needle) or vial (original vial provided by the manufacturer of Velcade) at scheduled times and diluted with 0.9% sodium chloride injection solution (0.9% sodium chloride, USP, B. Braun Medical S.A., Rubí, Spain, batch number: 20FIA013) to an expected bortezomib concentration of 125 µg/mL.

The bortezomib concentration in each sample was determined by interpolation from the standard curve.

The degradation product levels were determined by a response factor, which is the ratio between the signal produced by an analyte and the quantity of analyte that produces the signal. Ideally, and for easy computation, this ratio is unity (one). In real-world scenarios, this is often not the case.

Degradation product levels were measured by comparing the analytical response of the degradation product to that of the reference standard (Q 3 B (R2) Impurities in New Drug Products)^32^.

Assays were performed immediately after the samples were withdrawn.

#### Assay validation

Assay validation was conducted following official requirements based on the International Conference on Harmonization (ICH) guidelines and document Q2(R1)^48^.

For bortezomib, the linearity of the method was evaluated on 3 different days at 6 concentrations (50, 75, 100, 125, 150 and 175 µg/mL), and the linearity was determined by least squares linear regression analysis of the peak areas of bortezomib versus the concentration of the bortezomib standards. The precision of the method was assessed by evaluating the interday and intraday RSDs of samples at 125 µg/mL (n = 6 denotes 6 times the average of 2 injections). For interday RSD evaluations, 2 samples (125 µg/mL) were injected in duplicate on 3 different days.

#### Stability-indicating nature of the HPLC method

The stability-indicating nature of the HPLC method should be tested to ensure that the degradation products do not interfere with the measurement of the drug concentration in the solutions. To determine the stability of the assay, solutions containing the drug and its degradation products were analysed.

**Figure 4:**
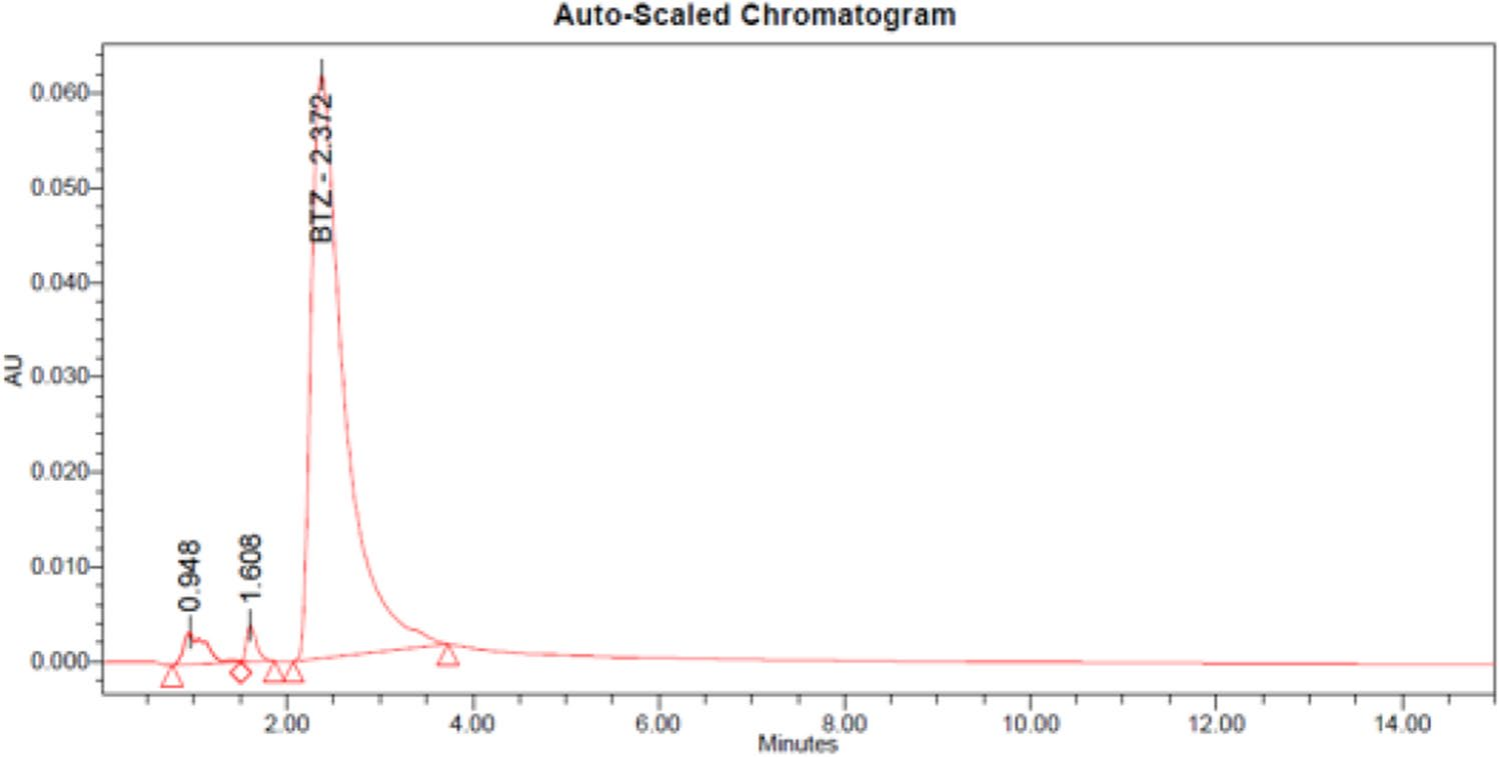
Chromatogram of a heat-treated bortezomib sample 60 °C for 1 h).

Then, the solutions of bortezomib were subjected to stress degradation using acid (1 N hydrochloric acid, Sigma‒Aldrich, E7144), base hydrolysis (1 N NaOH, Sigma‒Aldrich, 655,104), oxidation (0.3% hydrogen peroxide, Sigma‒Aldrich, H1009), and heat (60 °C) (Fig. 4). The sample was analysed every 30 min until approximately 20% of the peak corresponding to bortezomib disappeared, at which point the degradation products were detected. The resulting chromatograms were compared with chromatograms obtained from a freshly reconstituted solution of bortezomib (2.5 mg/mL in 0.9% sodium chloride). Each chromatographic run was approximately 30 min for the detection of the possible degradation products eluted before and after the bortezomib peak^35–37,43–45^.

#### Impurities inspection: degradation products of the reconstituted solution

The quantitation limit for the analytical procedure should not be more than ( ≤) the reporting threshold for the unidentified degradation products (0.10% of the drug substance in the drug product)^32^.

Degradation product levels were measured by comparing the analytical response for a degradation product to that of the cytotoxic drug standard^32^.

The purity of the mean peak was evaluated using a photodiode array (PDA) detector.

#### Mass balance

The mass balance was monitored according to ICH requirements^24^ based on the following definition: the process of adding together the assay value and degradation product levels to determine how closely these add up to 100% of the initial value, with due consideration of the margin of analytical error and other aspects such as the molar attenuation coefficient.

An acceptable mass balance value means that the experimental values are suitable for the determination of the chemical stability^24^.

### Physical inspection

The solution appearance and colour needed to be assessed throughout the stability study by visual inspection^49^ of samples withdrawn from the containers. Its evaluation was performed in a systematic and standardized way, incorporating more sensitive and specific techniques that complement visual control.

In the present work, the particle size of freshly reconstituted and stored reconstituted preparations (vials and syringes) was measured with a laser diffraction particle size analyser, considering the medium of the samples (water or/and reconstituted solution matrix). For this case, the samples were analysed using a Microtrac^®^ SRA 150 Laser Diffraction Particle Size Analyser, U.S.A. (measuring range 0.7 µm to 700 µm)^48^. Since Velcade contains 35 mg of mannitol, a blank sample was prepared with mannitol (Merck, M0200000). The reference sample was polystyrene latex beads with a narrow size distribution and a certified particle diameter (EPRUI Biotech Co., Ltd.). The measurement duration was 60 s. Particle size assays were performed immediately after the samples were collected.

When particles ≥ 10 µm were not detected for the analysed samples (freshly reconstituted samples and stable samples), the potential particle growth needed to be monitors, especially if the reconstituted solution was stored in a refrigerator due to the possibility of precipitation of subvisible particles. Then, the histograms obtained by dynamic light scattering^48^ from freshly and stored reconstituted products (vials and syringes) were compared. For this case, the samples were analysed by dynamic light scattering using Microtrac MRB’s NANOTRAC Flex, U.S.A. (measuring range 0.3 nm—10 µm)^48^.

For both Microtrac^®^ SRA 150 Laser Diffraction Particle Size Analyser and Microtrac MRB’s NANOTRAC Flex, information regarding the limit of detection (the minimum number of particles needed to be present in the solution to be detected) was unavailable; this is a limitation of our study.

### Physicochemical inspection

The pH is monitored.

At each time point, samples were withdrawn from the containers for pH measurements (HANNA instruments, HI5522 and HANNA calibration solutions). Variations of more than one unit of the pH value were interpreted^23,50^.

### Data analysis

Statistical analyses were performed using Statgraphics^®^ software (Statgraphics 19®centurion, Statgraphics Technologies, Inc. & StatPoint Technologies, Inc., Warrenton, VA, USA).

The results are expressed as the mean ± S.D.

Statistically significant differences were determined by one-way analysis of variance (ANOVA) following the least significant difference (LSD) post hoc test.

The initial cytotoxic drug concentration was set at 100%, and subsequent sample concentrations are expressed as a percentage of the initial concentration.

According to the ICH, “significant change” for a drug product is defined as a 5% change in assay from its initial value and/or as failure to meet the acceptance criteria for appearance and physical attributes (e.g., colour)^24^. It is recommended that the stability end point should not be lower than 95% of the initial concentration (T-95), especially in the case of anticancer drugs with a low therapeutic index and specific administration routes of intrathecal, ocular, intravenous or intra-arterial^2,4,24^.

The approved specification limit for the assay of bortezomib in Velcade^®^ is reported to be 90.0–110.0% in the USA and 95–105% in Europe^6,7^. The recommended end point of the proposed protocol was applied; the chemical stability of the bortezomib solution was defined as the retention of ≥ 95% of the initial drug concentration with a 95% confidence interval.

From a microbiological point of view, the method of opening/reconstitution/dilution prevents the risk of microbial contamination. This method was performed under controlled and validated aseptic conditions^25,51^. Moreover, the microbiological stability of aseptic preparations from hospital pharmacies was not evaluated in this study. A previous study showed that reconstituted vials of bortezomib were microbiologically stable for at least 11 days under class IIB cabinet working conditions^51^.

## Data Availability

The datasets used and/or analyzed during the current study available from the corresponding author on reasonable request.
